# Development of a microalgal peloid for thermotherapeutic uses

**DOI:** 10.1007/s00484-025-02967-8

**Published:** 2025-07-16

**Authors:** M. Lourdes Mourelle, Carmen P. Gómez, M. C. Martín de la Cruz, Gonzalo Cortés, Ignacio Cortés, José L. Legido

**Affiliations:** 1 FA2 Research Group, Department of Applied Physics, University of Vigo, Campus Lagoas-Marcosende s/n, 36310 Vigo, Spain; 2 CIM_ECIMAT. University of Vigo, Illa de Toralla s/n, 36331 Vigo, Spain; 3 Balneario El Raposo, Raposo, s/n, 06392 El Raposo, Badajoz, Spain

**Keywords:** Peloid, *Monoraphidium pusillum*, Thermophysical properties, thermotherapy

## Abstract

Peloids are therapeutic agents used in thermal medical / spa centers for the treatment of several ailments, mainly rheumatological diseases, sport injuries and dermato logical disorders. Peloids are composed by a mixture of a solid phase (clay, silt, peat, etc.), a liquid phase (mineral-medicinal water or seawater/salt-lake water), and a biological phase which include cyanobacteria and/or microalgae. This work describes the process of formulating a peloid from mineral-medicinal water (Balneario El Raposo), a solid clayey substrate, a microalga (*Monoraphidium pusillum*), and rosmarinic acid for use in muscular-skeletal disorders. In order to evaluate its thermotherapeutic potential, thermophysical properties have been studied. The development of a “phyto-peloid” could be of interest in order to improve the anti-inflammatory properties of thermal muds.

**Supplementary Information:**

The online version contains supplementary material available at 10.1007/s00484-025-02967-8.

## Introduction

Peloids are mixtures of clays, sediments or peat with mineral-medicinal water, sea or salt-lake water that are used in spa therapy for different treatments, mainly rheumatological diseases, sport injuries and dermatological disorders.

Peloids have been named and defined by different authors along the time. Judd Lewis, President of the “International Standard Measurements Committee proposed to give a generic name and classify the “Peloids”, comprising other denominations in different countries and languages as Boue, Fango, Gyttya, Limon, Lutum, Moor, Peat, Sapropel, Schlick, Seaweed, or Torf. Thus, the Greek word “Pelòs” (πελδς) was agreed at the Wiesbaden Congress (Germany) of the International Society of Medical Hydrology (ISMH) in 1938 (Maraver Eyzaguirre 2006).

In this congress, definition of peloid was achieved and adopted by the International Peloids Committee, being as follows: “Peloids are substances that are formed in nature through geological processes and that when finely granulated, mixed with water, have applications in medical practice in the form of baths or poultices” (Gomes et al. 2021).

The concept of peloids evolved and in 2013 the Working Group led by Gomes defined peloids as “Peloid is a maturated mud or muddy dispersion with healing and/or cosmetic properties, composed of a complex mixture of fine-grained natural materials of geologic and/or biologic origins, mineral water or sea water, and commonly organic compounds from biological metabolic activity” (Gomes et al. 2013).

Peloids can be natural or prepared “ad hoc” from clays, sediments or silts, mixed with mineral-medicinal water; and can be also classified as inorganic, organic or mixed; with maturation process (natural or artificial) (Gomes et al. 2013), or extemporaneous (that is, prepared and applied immediately without undergoing a maturation process) (Legido et al. 2007).

Peloids are composed by a mixture of a solid phase (clay, silt, peat, etc.), a liquid phase (mineral-medicinal water or seawater/salt-lake water), and a biological phase which include cyanobacteria and/or microalgae. All components are relevant: solid phase which contributes to the heat exchange ability; liquid phase which contains minerals and trace elements of great relevance to improve anti-inflammatory properties, and also contributes to the heat exchange ability; and biological phase that have been shown to provide compounds with biological action also linked to therapeutic properties (Carretero 2020a, b).

Peloids, in combination with mineral-medicinal water, are used for the treatment of various rheumatological diseases, including osteoarthritis and musculoskeletal ailments, which are accompanied by pain (Rapolienė et al. 2020), exerting anti-inflammatory, antioxidant and chondroprotective actions (Cozzi et al. 2018; Fioravanti 2020; Gálvez et al. 2018, 2024; Cheleschi et al. 2022; Ortega-Collazos et al. 2024).

Different microalgae and cyanobacteria can be found in peloids, either naturally or ad hoc prepared. In ad hoc prepared peloids, this biological/microbial phase is cultivated and added to the mixture of solid substrate and medicinal mineral water for later maturation or extemporaneous use (Mourelle et al. 2021, 2024). Some examples are Euganean thermal muds (Gris et al. 2020), and Balaruc-les-Bains Thermal muds (Duval et al. 2021), in which biological compound form microalgae have been shown to exert anti-inflammatory activities (Zampieri et al. 2020, 2022; Demay et al. 2020).

In this scenario, some research focuses on microorganisms found in fresh and thermal water that could be of interest in pelotherapy; that is the case of *Monoraphidium* sp. which, despite being poorly studied, is investigated for its poly-unsaturated fatty acids contain (Vinitha et al. 2022). Specifically, *Monoraphidium pusillum* has a high lipids content, and it is easy to grow (Hawrot-Paw et al. 2020; Bácsi et al. 2022). Furthermore, *Monoraphidium* sp. strains are being investigated to obtain carotenoids and lipids for the pharmaceutical and nutraceutical applications (Yadav et al. 2023).

Rosmarinic acid is a phenolic compound commonly found in the Lamiaceae plant species as rosemary, lavender, oregano, savory, etc., that has been shown to possess antioxidant and anti-inflammatory properties. Rosmarinic acid has been used in folk medicine and phytotherapy with several pharmacological activities and its anti-inflammatory properties, mainly in arthritis and other osteoarticular ailments (Rocha et al. 2015; Mahmoud et al. 2021).

In rheumatic diseases, rehabilitation and other osteo-muscular problems, peloids are applied hot or warm, with the thermophysical properties being one of the most important aspects to take into account when formulating and evaluating these thermal products (Legido et al. 2007; Rebelo et al. 2011; Carretero 2020a, b).

Thermophysical properties play a very important role in thermotherapeutic applications (Gomes et al. 2013; Carretero 2020a, b; Maraver et al. 2021), and specifically in the treatment of musculoskeletal disorders, since they are the basis of energy exchanges between the peloid and the body, in all its forms of use (poultices, wraps and baths). Thus, density and specific heat allow obtaining the volumetric heat capacity that informs of the amount of energy stored in a peloid per unit of volume when increasing its temperature; on the other hand, the thermal conductivity of a peloid measures the capacity to transmit heat by conduction; the quotient between thermal conductivity and volumetric heat capacity provides the value of thermal diffusivity that offers the speed with which heat is diffused in a peloid. In recent years, much progress has been made in the study of the thermophysical characterization of peloids, so that today it is possible to obtain data on the thermophysical characteristics of different peloids for pelo-therapeutic applications.

One of the first studies of peloid properties was carried out by Lewis in 1935 (Lewis 1935); later Berbenni 1960 (Berbenni 1960), and Prat and Brozeck in 1963 (Prat and Brozeck 1963) provided new data. The first quantitative data were published by Ferrand and Yvon in 1991 (Ferrand and Yvon 1991); Cara et al. in 2000 (Cara 2000) provided data on peloids from the Sardinia area in Italy, and Beer et al. in 2003 (Beer et al. 2003) in Bulgaria; other classic articles that provided important data are those carried out by Veniale et al. in 2004 (Veniale et al. 2004) and Legido et al. in 2007 (Legido et al. 2007), in which a study of the thermal behavior of various materials for pelotherapy uses was carried out. In addition, Rebelo et al. (Rebelo et al. 2011) presented data on physical properties of Portuguese peloids, and Pozo et al. (Pozo et al. 2013) of samples of different Spanish peloids. On the other hand, Casas et al. (Casás et al. 2013) presented data of saline peloids, and Knorst-Fouran et al. (Knorst-Fouran et al. 2012) data of the Terdax peloid from Dax (France). Caridad et al. (Caridad et al. 2014) measured the densities and thermal conductivities of materials for their possible pelotherapy applications, and Carretero et al. (Carretero et al. 2014) provided the thermophysical properties of mixtures of clays with mineral-medicinal waters. Likewise, Sánchez Espejo et al. (Sánchez-Espejo et al. 2014) determined the specific heat of peloids matured with water from Graena (Spain), Khiari et al. (Khiari et al. 2014) provided the values ​​of some peloids from Tunisia, Karakaya et al. (Çeli̇K Karakaya et al. 2017) those of some peloids from Turkey, and Mato et al. (Mato et al. 2017) carried out a study of specific heats of a peloid with kaolin. Other interesting studies are those on marine peloids by Glavas et al. (Glavaš et al. 2017), who determined thermophysical properties of saline peloids from Slovenia. On the other hand, Pozo et al. (Pozo et al. 2019) provided new data on peloids from mixtures of clay and water, while Özay et al. (Özay et al. 2020) presented data on physical properties of peloids from Turkey, and, in 2021, Fernández-González et al. (Fernández-González et al. 2021) showed the thermal behavior of peloids made with water from Lanjarón in Granada (Spain). Later on, Gankhurel et al. (Gankhurel et al. 2021) presented data on peloid density from Mongolia, and Maraver et al. (Maraver et al. 2021) published the thermal properties of various peloids from around the world. And recently, Akimzhanova et al. (Akimzhanova et al. 2024) showed the heat capacity behavior of peloids from Kazakhstan lakes.

Furthermore, considering that the main effect of peloids is anti-inflammatory, it may be of interest to enhance this effect by adding compounds with recognized anti-inflammatory power derived from essential oils, such as rosmarinic acid (Gautam et al. 2019).

The aim of the study was focused on the design and development of a peloid made of mineral-medicinal water and microalgae, to which rosmarinic acid has been added to increase its possible thermotherapeutic effect; the characterization of the different phases and components is described as well as the procedure to prepare the peloid. To confirm this assumption, the thermophysical properties are studied and, in a separate study, its effectiveness in patients with osteoarthritis has been investigated, focused on evaluating its impact on the innate immune response (Ortega-Collazos et al. 2024).

## Materials and methods

El Raposo peloid is composed by a mixture of sulfate mineral-medicinal water, a clayey-silt solid phase, and a microalga obtained through growth in bioreactors; after maturation, rosmarinic acid has been added to this mixture with the purpose of increasing its anti-inflammatory properties.

### El Raposo soil

The “El Raposo soil” comes from the land of the area where the thermal spa is located and has been used since its opening 100 years ago to prepare peloids. The soil was subjected to a grinding process and then sifted before mixing with thermal spring water. The characteristics of the soil were analyzed at the Scientific and Technological Support Centre (C.A.C.T.I.) of the University of Vigo. The mineralogical analysis was carried out using X-ray diffraction, which studies the deviation of an X-ray beam when it hits the sample. The deviation allows the identification of crystalline compounds.

Table 1 shows the results obtained on the crystalline phases that make up the El Raposo soil, where it can be seen that it is mainly composed for quartz and calcite.

**Table 1:** Crystalline components of El Raposo soil

Crystalline phase	Chemical formula	SemiQ. (%)
Quartz	SiO_2_	41.8
Calcite	CaCO_3_	48.6
Mica/Muscovite	(K, Na) (Al, Mg, Fe)_2_ (Si3.1, Al0.9)_4_ O_10_ (OH)_2_	2.3
Albite	NaAlSi_3_O_8_	4.9
Vermiculite	Mgx (Mg, Fe)_3_ (Si, Al)_4_ O_10_ (OH)_2_· 4H_2_O	1.1
Kaolinite	Al_2_Si_2_O_5_(OH)4	1.3

In addition, an organic elemental analysis was carried out, determining the elements N, C, H and S in the El Raposo soil. This determination was made by burning the sample in a furnace at 1.000 °C. The gases generated are pumped with helium through reagents that reduce the number of gaseous species present in the mixture resulting from the combustion. They are then passed to a LECO CN 2000 gas chromatograph. Table 2 shows the results obtained from El Raposo soil, in which it can be observed the percentages of nitrogen, carbon, hydrogen and sulfur in the sample.

**Table 2:** Elemental analysis of El Raposo soil

% *N*	%C	%H	%S
0.16	3.70	0.72	< 0.30

An inorganic elemental analysis was also carried out using X-ray fluorescence (XRF) with the Siemens SR300 equipment that uses the secondary or fluorescent emission of X radiation generated by exciting a sample with an X radiation source, which allows the quantitative determination of the elements of the El Raposo soil. The results of the analysis are shown in Table 3, where it can be observed that the soil is mainly composed of the elements silicon and calcium, also containing aluminum, iron, potassium and magnesium.

**Table 3:** Inorganic elemental analysis of El Raposo soil

Chemical element	Concentration %
Na	0.178
Mg	1.441
Al	9.262
Si	22.344
P	0.096
S	0.026
K	2.358
Ca	14.65
Ti	0.629
Cr	0.0109
Mn	0.1007
Fe	4.183
Ni	0.0060
Cu	0.0067
Zn	0.0129
Sr	0.0093
Zr	0.0212
Ba	0.0896

**Fig. 1:**
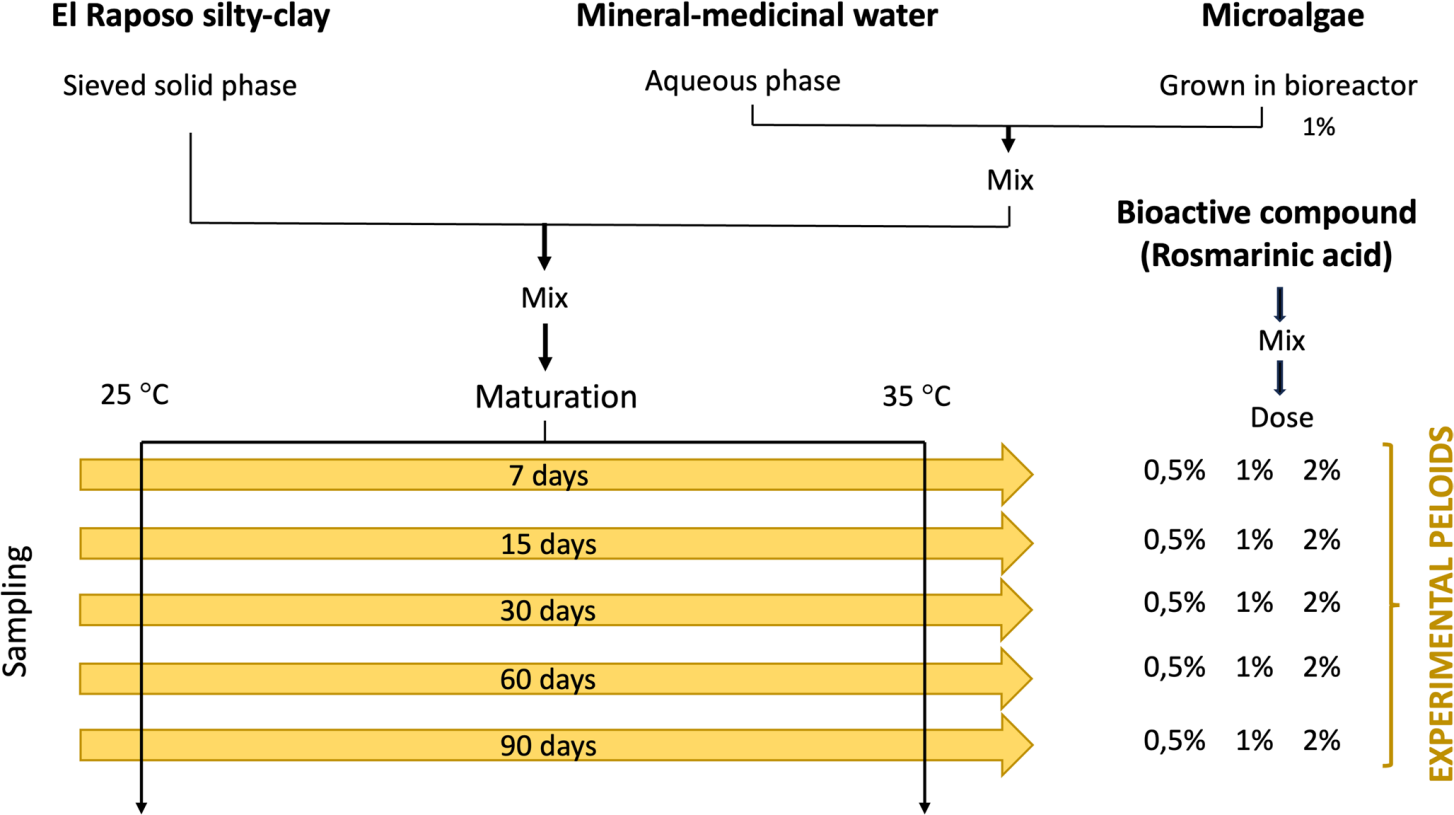
Process for obtaining experimental peloids

Supplementary Figure 1 shows the electron microscopy images of El Raposo soil taken using a JEOL JSM 6700 F scanning electron microscope (Suplemmentary Figs. 1a, b,c).

### El Raposo mineral-medicinal water

The characteristics of El Raposo mineral-medicinal water were analyzed at the Scientific and Technological Support Centre (C.A.C.T.I.) of the University of Vigo, at the Research Support Service (S.A.I.) of the University of A Coruña, and at the Applied Physics Laboratory of the University of Vigo.

Table 4 shows the physical-chemical parameters studied. The pH was determined using the Crison pH-meter Basic 20+; the electrical conductivity was determined using the Mettler Toledo, Seven compact; the density was determined using an Anton Paar DMA 4500 mechanical oscillation densitometer and the dry residue was obtained by subjecting the sample to 110 °C for 24 h.

**Table 4:** Physical-chemical parameters of El Raposo mineral-medicinal water

Parameter	Result	Parameter	Result
**pH (26**,**6 °C)**	8.05	**Dry Residue**	800 mg/L
**Density (20 °C)**	0.9986 g/cm^3^	**Density (25 °C)**	0.9975 g/cm^3^
**Electrical Conductivity (20 °C)**	0.802 mS/cm	**Electrical Conductivity (25 °C)**	0.900 mS/cm

Table 5 shows the chemical elements and hardness of the mineral-medicinal water of the El Raposo Thermal Spa, carried out using the Thermo Finnigan ELEMENTXR Magnetic Sector ICP-MS method.

**Table 5:** Chemical elements, other compounds and hardness of El Raposo mineral-medicinal water

Element	mg/L	Element	µg/L
Na^+^	55	Mn	12
Mg^+2^	19	Fe	< 5.0
Si	15	Li	6
Ca^+2^	122	Al	16
K^+^	2	Ag	< 2.5
Cl^-^	75	Ba	350
SO_4_^−2^	25	Cd	< 1.5
HCO_3_^-^	400	Cr	< 5.0
Be	< 2.5	Cd	< 1.25
Co	< 2.5	Cr	< 5.0
Cu	< 5.0	Hg	< 0.25
Ni	< 5.0	Pb	< 2.5
Rb	< 2.5	Se	< 2.5
Sr	229	Zn	27
**Compound**	**mg/L**	As	< 2.5
CaCO_3_ (hardness)	394	Be	< 2.5
SiO_2_	32.4	Co	< 2.5
		Cu	< 5.0
		Ni	< 5.0
		Rb	< 2.5
		Sr	229

### Biological phase

**Fig. 2:**
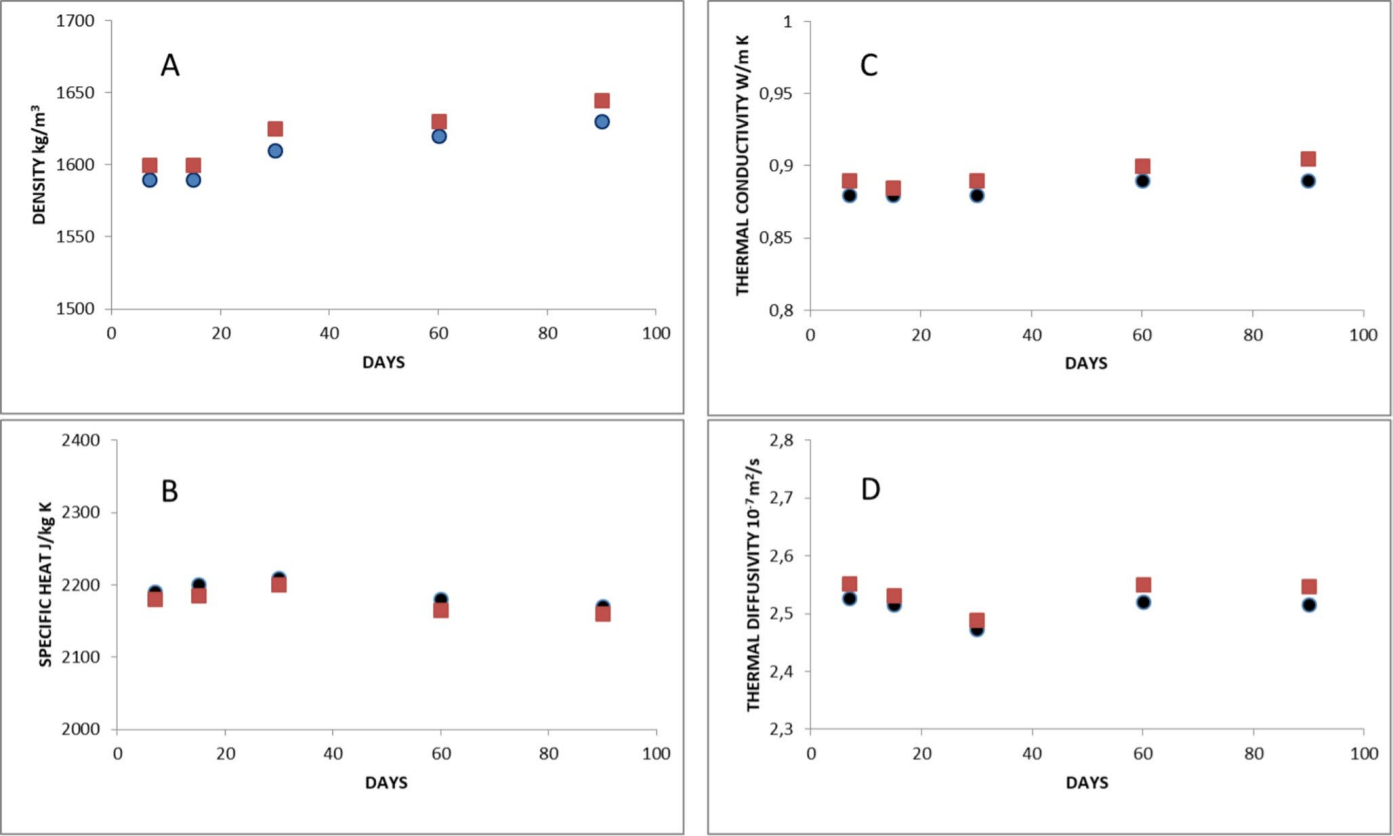
Thermophysical properties behavior versus maturation time. **A**, Density; **B**, Specific heat; **C**, Thermal conductivity; **D**, Thermal diffusivity. (Blue circles 35 °C; red squares 25 °C)

The biological phase of the new peloid is a microalga from a pond at the El Raposo Thermal Spa facilities). The samples were collected in sterile containers and kept in the best conditions during transport using a 0.2-micron filter that allowed air to enter. Figure 2 shows a sample of the algae from the area on a Petri dish (Supplementary Fig. 2).

**Fig. 3:**
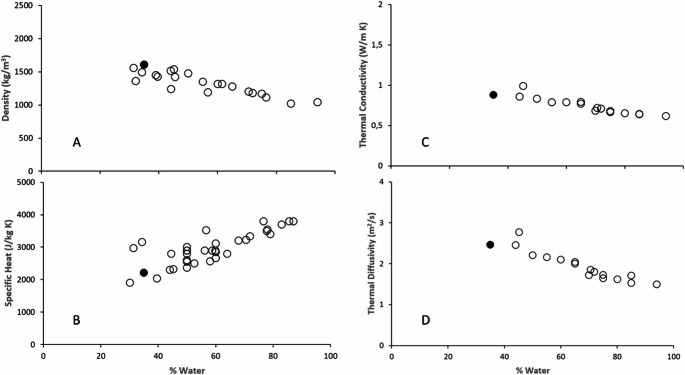
Comparation of obtained data from microalgal peloid and other studied peloids. **A**, Density; **B**, Specific heat; **C**, Thermal conductivity; **D**, Thermal diffusivity. Black circles data from this work and white circles from the bibliography

After receiving the samples, an initial microscopic analysis was carried out. A Nikon Eclipse 90i fluorescence microscope with image analysis (Nikon refrigerated 5 Mpx camera) belonging to the Toralla Marine Science Station (ECIMAT) of the University of Vigo was used. The images were obtained using bright field microscopy. Figure 3 shows the species observed in the initial samples, and Fig. 4 shows the selected species; the length measurement is 26.98 microns (Supplementary Figs. 3 and 4).

The microalgae were sent to the Spanish Algae Bank (BEA) requesting a morphological identification report and a molecular identification report, using DNA sequencing.

According to the Spanish Algae Bank report, the morphological identification identifies the strain as *Monoraphidium pusillum* (Printz) Komárková – Legnorová, belonging to the Chlorophyceae class, Sphaeropleales order and Selenastraceae family. Regarding the molecular identification, the strain is identified as *Podohedriella* sp.

To determine the purity of the DNA sample, the quotient between the absorbance values ​​at 260 and 280 nm (A 260/A 280) is calculated. A DNA sample of acceptable purity must present an A 260/A 280 = 1.8. The value of this ratio in the molecular identification was 1.76.

Both reports place the strain in the Class Chlorophyceae, Order Sphaeropleales and Family Selenastraceae. Regarding genus and species, the morphological study identifies it as *Monoraphidium pusillum* and the phylogenetic analysis identifies it as *Podohedriella* sp. The strain is located in the *Podohedriella* clade, with 60% similarity to *Podohedriella falcata* (based on the Maximum “Likelihood” and “MrBayes” methods). In the phylogenetic tree, it is observed that *Monoraphidium* is a polyphyletic genus and that *Podohedriella* is found sharing a clade with *Monoraphidium* species. In particular, this is a group that is under taxonomic review, so more extensive phylogeny work would be necessary.

Initially, each cell separated by sorting was deposited in one of the 48 wells of a sterile (untreated) FALCON BD cell culture plate containing sterile culture medium. Growth in the wells of the plate was monitored using a Nikon model TE2000S inverted microscope (ECIMAT, University of Vigo). Cultures that, after two weeks, again showed greater and faster growth were inspected under a microscope (by their morphology under the microscope) the same type of cells growing in said wells.

Following microscopic inspection, the contents of each individual well were aseptically transferred to a sterile 25 cm^2^ culture flask containing culture medium (Tables 6 and 7). Following growth, the entire volume of the 25 cm^2^ flask was used to initiate culture in a sterile flask (also fitted with an air filter stopper) with a growth area of ​​75 cm^2^.

**Table 6:** Quantities required for the Preparation of the culture medium

Chemical compound	Quantity
Ca(NO_3_)_2_	0.2 g/L
MgSO_4_	0.1 g/L
KH_2_PO_4_	0.05 g/L
Solution C (GoldMedium-FWS)	50 µL/L

**Table 7:** Quantities required for the Preparation of the culture medium (modification of quantities but same compounds as those required for the Preparation of goldmedium Fresh-Water Species)

Chemical compound	Quantity
Mixture of NaNO_3_ and KH_2_PO_4_	0.258 g/L
MgSO_4_ ∙ 7H_2_O	0.075 g/L
CaCl_2_ ∙ 2 H_2_O	0.025 g/L
Premix Trace elements and vitamins Goldmedium	0.00725 g/L

The culture medium was subjected to a sterilization process by autoclaving at 121 °C for 30 min. These aliquots with added culture medium were kept at room temperature in sterile culture flasks with a growth area of ​​25 cm^2^ that have a filter with a 0.2-micron hydrophobic membrane to allow gas exchange. The lighting cycle was 12 h of light/12 h of darkness with LED lighting of 850 lumens (10 W of power). After the initial growth observed, the procedure was repeated again, this time taking aliquots of these growing cultures. Figure 5 shows the microalgae in the growth phase (Supplementary Fig. 5).

Growth studies were also carried out after maintaining the microalgae at temperatures of 4 °C and 40 °C. In both cases, the subsequent growth of the microalgae at room temperature is similar to those carried out without subjecting the microalgae to low and high temperatures. The microalgae culture was subjected to a continuous centrifugation process, separating the concentrated fraction from the diluted phase. The result of this centrifugation is a concentrated pure microalgal paste.

The chemical analysis of the powder obtained from the microalgae paste has been carried out from the SEM images taken at the CACTI of the University of Vigo **(**Table 8).

**Table 8:** Percentage of the constituent elements of the microalgae

Chemical element	Concentration %
C	72.22
O	21.70
Na	0.19
Mg	0.46
Si	0.34
P	1.68
S	0.96
Cl	0.12
K	0.89
Ca	1.44

For the preparation of the peloids, the microalgae growth was carried out in 80-litre vertical plastic bags (photobioreactor), located at the El Raposo Thermal Spa. Given the ideal climatic conditions of the area, these were located in an area outside the thermal spa with a pH control system by injection of air and CO_2_.

### Rosmarinic acid

Rosmarinic acid was supplied by the company Sigma-Aldrich, CAS number 20283-92-5, whose appearance is powder and dark brown in color.

### Peloid Preparation

In this phase, different peloid preparation processes were developed, in order to standardize the process and evaluate which are the best conditions that maintain all the therapeutic properties of the different products generated.

The starting point was the sifted solid substrate and subjected to a drying process in an oven at 110 °C for 24 h. The microalgae were incorporated in the aqueous phase, as obtained in the photobioreactor, with a concentration of 1%. Once both solid: liquid phases were mixed (approximate ratio 65:35), the maturation processes were carried out.

The maturation processes were carried out at two temperatures (25 °C and 35 °C) and were subjected to a daily stirring process. Sampling was carried out at 7, 15, 30, 60 and 90 days. The bioactive compounds were added to each type of sample. Bioactive compounds should not be mixed before the maturation process as they may be modified during the process. The proportions of the fractions of bioactive compounds (rosmarinic acid) added will be: 0.5%, 1% and 2%.

Figure 1 shows a diagram of the preparation process of the different experimental peloids.

### Methods for determining thermophysical properties

#### Density

To determine the density of the mixtures, a pycnometer (Álamo, 25 ml) (Caridad et al. 2014) is used and n-hexane (Sigma Aldrich, 99% purity) as a reference liquid.

#### Specific heat

The experimental specific heat is determined using a Calvet microcalorimeter equipped with a device that allows operation in the absence of the vapor phase and using a calorimeter cell with a volume of approximately 10 cm^3^. The temperature is controlled using a digital thermometer and regulated for an accuracy of 0.01 K. The equipment is connected to a Philips PM2532 multimeter (Glavaš et al. 2017) that allows the detection of very small signals (microvolts). The determination of the specific heat is based on the method described by Calvet and Prat in 1963 (Calvet and Prat 1963), from the thermograms produced by supplying a small power with the Setaram EJP 30 Power Supply in the measuring cell, which by the Peltier effect produces a small variation in temperature, during which the specific heat can be considered constant. The method is described in Paz Andrade et al. (Paz-Andrade et al. 1970), by measuring the area that occurs in the cooling curve, determining the calibration constants using known specific heat standards.

#### Thermal conductivity

This equipment is used to measure thermal conductivity, the measurement principle of which is based on the “Transient Line Heat Source” method. It includes a thermistor for reading temperatures and a microprocessor for data storage. The equipment consists of a reading unit and a thermal probe provided with a heating element and a thermoresistance, in accordance with the standards of ASTM D5334 and IEEE regulations 442–1981 (Pastoriza-Gallego et al. 2011).

#### Thermal diffusivity

Thermal diffusivity has been calculated from the data obtained from thermal conductivity, density and specific heat, according to the following expression (Casás et al. 2013):

$$\:\alpha\:=\frac{\lambda\:}{\rho\:cp}$$

where λ is the thermal conductivity, ρ is the density, and cp. is the specific heat capacity.

## Results and discussion

Once the results were analyzed, the maturation process at 35 °C was selected as it offers slightly better thermophysical results. Figure 2 A shows the behavior of density with maturation time, observing a stabilization of density, after 30 days, with an average value of 1630 kg/m^3^ at 35 °C; a slight increase in density is also observed as the temperature decreases (Fig. 2A).

The specific heat has a slight increase in the first days of ripening (Fig. 2B) and then decreases in the last days. The optimal value is found at around 2210 J/kg K.

Thermal conductivity increases slightly with maturation (Fig. 2 C), stabilizing at around 0.89 W/m K at 35 °C after 30 days. The behavior with temperature is a slight increase as the temperature decreases, with no significant variations in the value of thermal conductivity.

Figure 2 D shows the behavior of thermal diffusivity with respect to maturation. A minimum is observed at 30 days, which would indicate the optimal maturation of the peloid, with a value of 2.45 10^−7^ m^2^/s at 35 °C, which is a value similar to other peloids studied previously (Carretero 2020a, b). We can also observe a slight increase in thermal diffusivity as the temperature increases.

No significant variations have been detected in all thermophysical properties when adding rosmarinic acid.

It is well known that the thermotherapeutic actions of peloids are linked to their thermophysical properties, mainly heat capacity and thermal diffusivity and, therefore, to the ability to transmit energy in the form of heat to the tissues on which the peloid is applied (Legido et al. 2007). In the case at hand, the El Raposo peloid, the thermophysical and other properties of the natural peloid have been investigated by several authors (Maraver et al. 2021; Pozo et al. 2013; Armijo et al. 2016). The therapeutic effect has also been studied, comparing the natural peloid with a peloid produced by a controlled technological process (Gálvez et al. 2024). In this work, rosmarinic acid has been added to the peloid and its thermophysical properties have been studied to test the hypothesis of their improvement by the addition of rosmarinic acid.

Rosmarinic acid is a phenolic compound commonly found in the Lamiaceae plant species as rosemary, lavender, oregano, savory, etc. Rosmarinic acid is very well-known for its anti-inflammatory properties as several studies have shown that is able to suppress inflammation and angiogenesis (Rocha et al. 2015). In osteoarticular problems rosmarinic acid could also be useful; rosmarinic acid attenuates inflammation in experimentally induced arthritis in Wistar rats (Gautam et al. 2019); in vitro studies demonstrated the inhibitory effect on IL-6 production on catabolism induced by IL-1β in rat chondrocyte (Mahmoud et al. 2021), and also exerts antioxidant properties (Lu et al. 2023).

Different proportions of rosmarinic acid were tested to be added to the peloid (0.5%, 1% and 2%), observing no significant differences between them regarding the thermophysical properties of the peloid. Therefore, the proportion of 0.5% was chosen to avoid possible skin reactions.

Despite finding that the improvement of the thermophysical properties with the addition of rosmarinic acid is not significant, a clinical study was carried out to determine the possible improvements in the anti-inflammatory effects of the enriched peloid. Two peloids were compared, the above mentioned controlled-matured peloid, and the same supplemented with rosmarinic acid (Ros-A), finding that both treatments were effective in treating osteoarthritis, with significant improvements in pain (approximately 60%), function and quality of life, but the RosA-fortified peloid produced greater benefits, particularly in the anxiety/depression dimension of the EUROQOL questionnaire and in improving the immune response of neutrophils (Ortega-Collazos et al. 2024).

Additionally, it can be observed that the optimal maturation time of the peloid is one month, which agrees with other previous studies that establish that the changes in the composition of the peloids do not experience important variations after 3 months of maturation (Carretero 2020a, b; Bergamaschi et al. 2020).

When comparing the data obtained at 298.15 K with those in the literature (Legido et al. 2007; Rapolienė et al. 2020; Maraver et al. 2021; Lewis 1935; Berbenni 1960; Prat and Brozeck 1963; Ferrand and Yvon 1991; Cara 2000; Beer et al. 2003; Veniale et al. 2004; Pozo et al. 2013, 2019; Casás et al. 2013; Knorst-Fouran et al. 2012; Caridad et al. 2014; Carretero et al. 2014; Sánchez-Espejo et al. 2014; Khiari et al. 2014; Çeli̇K Karakaya et al. 2017; Mato et al. 2017; Glavaš et al. 2017; Özay et al. 2020; Fernández-González et al. 2021; Gankhurel et al. 2021; Akimzhanova et al. 2024) (Fig. 3), we observe a similar behavior with peloids that are used in pelotherapy treatments or were designed for that purpose. Thus, the density of 1610 kg/m^3^ is in the range of 1100 kg/m^3^ and 1800 kg/m^3^; the specific heat of 2210 J/kg K is a value that is in the average range of 2000 J/kg K and 3600 J/kg/K; the volumetric heat capacity of 3.5 MJ/m^2^ K is adequate for this type of treatments; the thermal conductivity of 0.89 W/m K is a normal value for peloids of predominantly inorganic solid phase (note that it is a difficult property to measure and there is not much reliable data). Finally, the thermal diffusivity of 2.45 10^−7^ m^2^/s can also be considered in the middle intervals of the mostly inorganic peloids.

## Conclusions

In order to improve the anti-inflammatory properties, a peloid has been designed and prepared by mixing the solid and liquid substrates with a microalga and, once the maturation process was completed, rosmarinic acid was added. The idea of ​​creating a “phyto-peloid” is not new, since peloids are traditionally mixed with plants (Nacakoğlu et al. 2020), but this is the first time that a compound with recognized anti-inflammatory activity such as rosmarinic acid has been added.

The results obtained from the thermophysical properties are not conclusive enough to evaluate its possible increase in thermotherapeutic capacity, but the clinical studies carried out to evaluate whether this new phyto-peloid has greater anti-inflammatory potential than the controlled maturation peloid of microalgae are conclusive, showing an improvement in the immune response of neutrophils, among other benefits (Ortega-Collazos et al. 2024).

## Electronic supplementary material

Below is the link to the electronic supplementary material.

Supplementary Material 1

Supplementary Material 2

Supplementary Material 3

## Data Availability

Data subject to third party restrictions.

## Declarations

### Disclaimer

The statements, opinions and data contained in all publications are solely those of the individual author(s) and contributor(s) and not of MDPI and/or the editor(s). MDPI and/or the editor(s) disclaim responsibility for any injury to people or property resulting from any ideas, methods, instructions or products referred to in the content.
