# Supplementary material for: Development of a microalgal peloid for thermotherapeutic uses
Source: Int J Biometeorol. 2025 Jul 16;69(9):2103–13. doi: 10.1007/s00484-025-02967-8 (PMC12479575; doi:10.1007/s00484-025-02967-8)
Supplement: Supplementary file 1 — Supplementary Material 1 [file 484_2025_2967_MOESM1_ESM.docx]

Supplementary Information

| 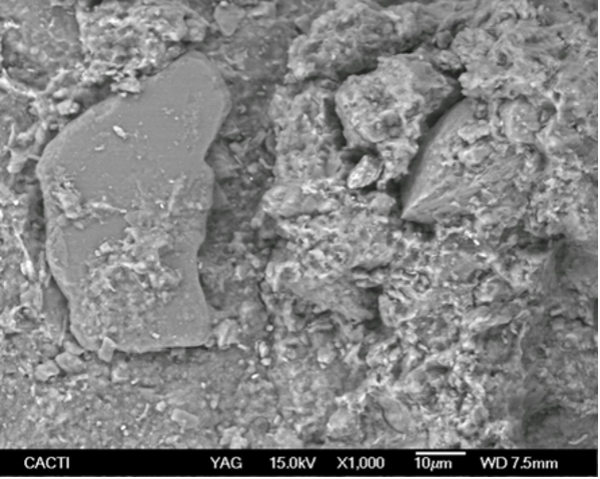  Figure 1a | 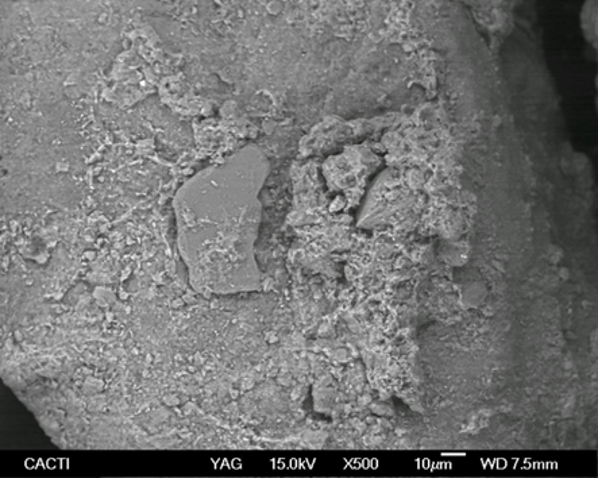  Figure 1b | 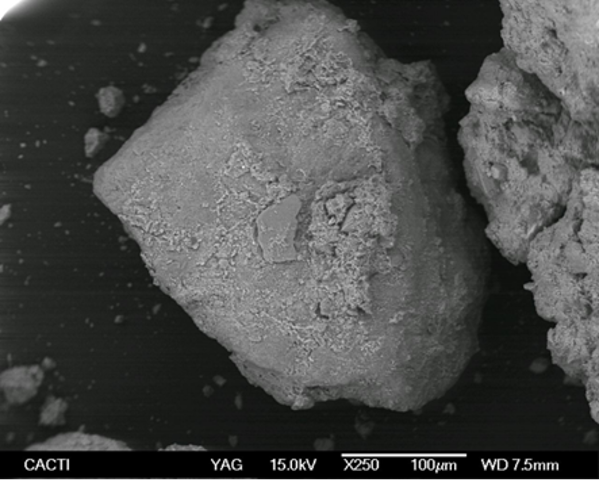  Figure 1c |
| --- | --- | --- |

**Figure 1.** Electron microscopy images of El Raposo soil at 1000, 500, and 250 magnifications.

| 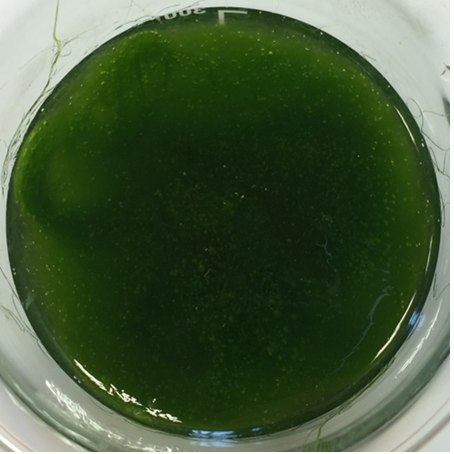  Figure 2 | 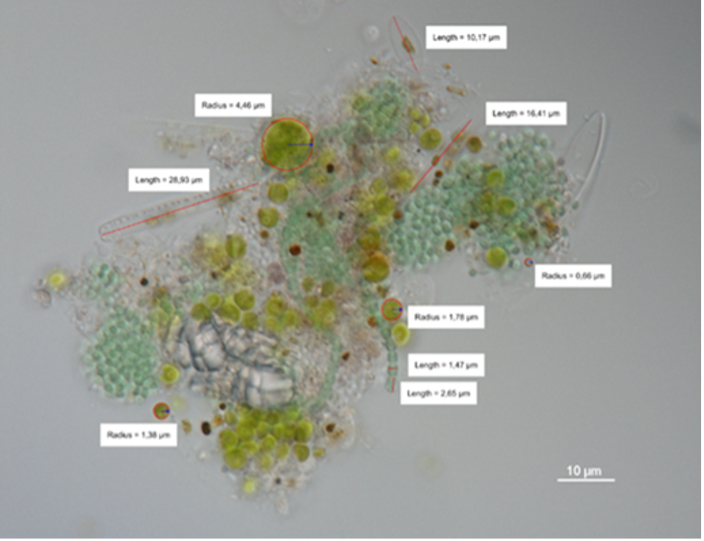  Figure 3 | 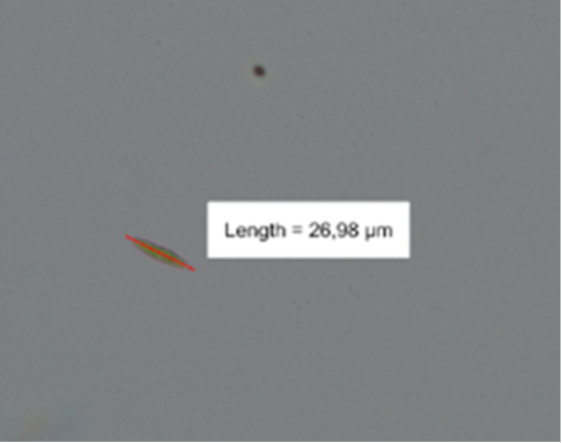  Figure 4 | 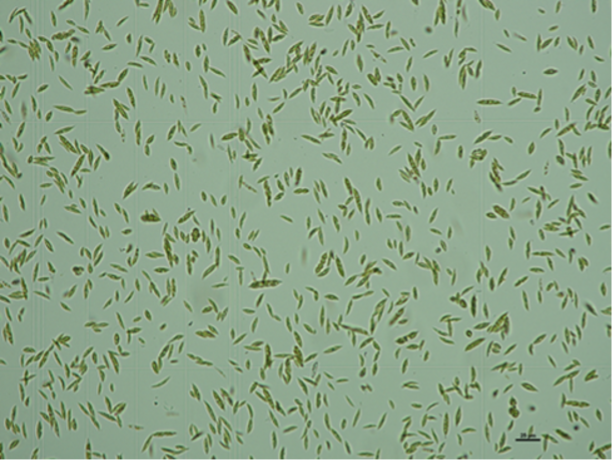  Figure 5 |
| --- | --- | --- | --- |

**Figure 2.** Microalgae from the pond at the El Raposo Thermal Spa.

**Figure 3**. Measurements (radius, length) of some of the species present in the sample with an immersion objective (planatic and apochromatic) of 100X and 1000 total magnifications).

**Figure 4.** Images obtained with 40X planar objective (10X eyepiece, 400x total).

**Figure 5.** Species in growth phase (image obtained with the Nikon Eclipse 90i microscope and with the 20X planar objective (200 total magnifications).
