# Supplementary figures and images for: Development of a microalgal peloid for thermotherapeutic uses
Source: Int J Biometeorol. 2025 Jul 16;69(9):2103–13. doi: 10.1007/s00484-025-02967-8 (PMC12479575; doi:10.1007/s00484-025-02967-8)

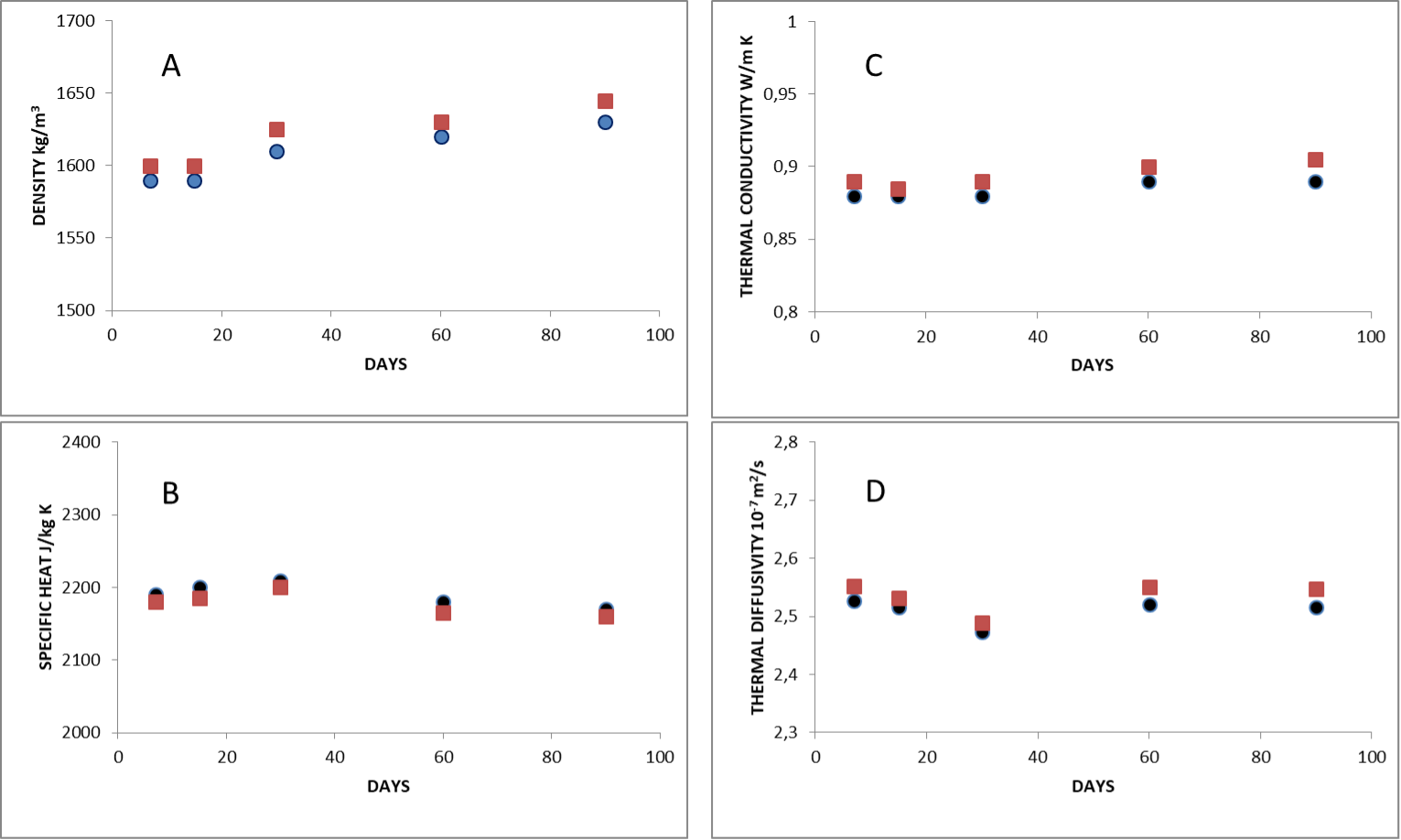

Supplement: Supplementary file 2 — Supplementary Material 2 [file 484_2025_2967_MOESM2_ESM.png]

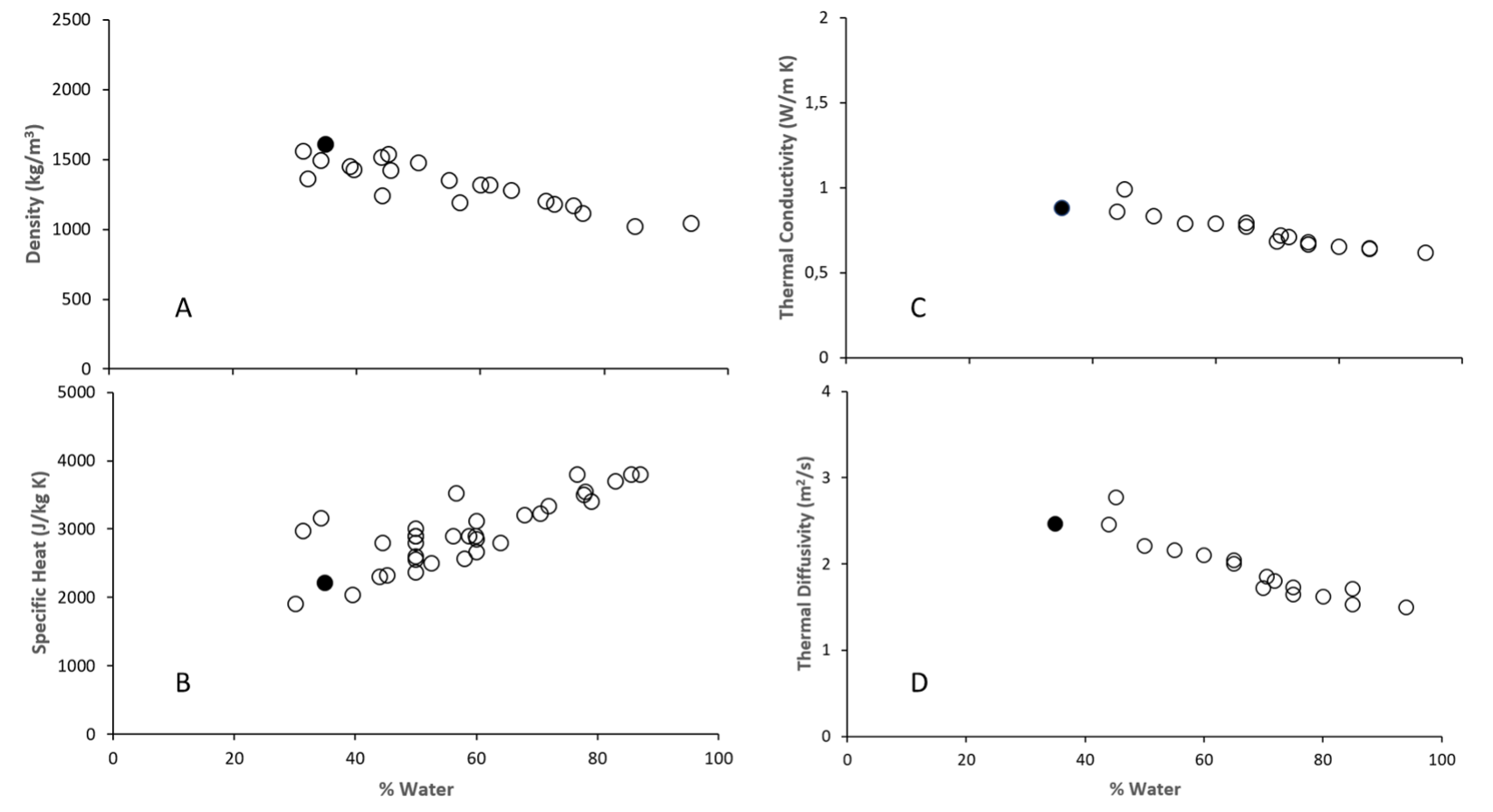

Supplement: Supplementary file 3 — Supplementary Material 3 [file 484_2025_2967_MOESM3_ESM.png]
